# Oscillator-Network-Based Ising Machine

**DOI:** 10.3390/mi13071016

**Published:** 2022-06-27

**Authors:** Yi Zhang, Yi Deng, Yinan Lin, Yang Jiang, Yujiao Dong, Xi Chen, Guangyi Wang, Dashan Shang, Qing Wang, Hongyu Yu, Zhongrui Wang

**Affiliations:** 1 Faculty of Engineering, The University of Hong Kong, Hong Kong 999077, China; u3008443@connect.hku.hk (Y.Z.); u3589550@connect.hku.hk (Y.D.); u3586466@connect.hku.hk (Y.L.); jiangeee@connect.hku.hk (Y.J.); yujiao_dong@hdu.edu.cn (Y.D.); cxishao@connect.hku.hk (X.C.); 2 School of Microelectronics, Southern University of Science and Technology, Shenzhen 518055, China; 3 Institute of Modern Circuit and Intelligent Information, Hangzhou Dianzi University, Hangzhou 310018, China; wanggyi@163.com; 4 Institute of Microelectronics, Chinese Academy of Sciences, Beijing 100029, China; shangdashan@ime.ac.cn

**Keywords:** oscillator network, Ising machine, combinatorial optimization, max-cut

## Abstract

With the slowdown of Moore’s law, many emerging electronic devices and computing architectures have been proposed to sustain the performance advancement of computing. Among them, the Ising machine is a non-von-Neumann solver that has received wide attention in recent years. It is capable of solving intractable combinatorial optimization (CO) problems, which are difficult to be solve using conventional digital computers. In fact, many CO problems can be mapped to finding the corresponding ground states of Ising model. At present, Ising machine prototypes based on different physical principles, such as emerging memristive oscillators, have been demonstrated, among which the Ising Hamiltonian solver based on the coupled oscillator network simultaneously holds the advantages of room-temperature operation, compact footprint, low power consumption, and fast speed to solution. This paper comprehensively surveys the recent developments in this important field, including the types of oscillators, the implementation principle of the Ising model, and the solver’s performance. Finally, methods to further improve the performance have also been suggested.

## 1. Introduction

**Figure 1:**
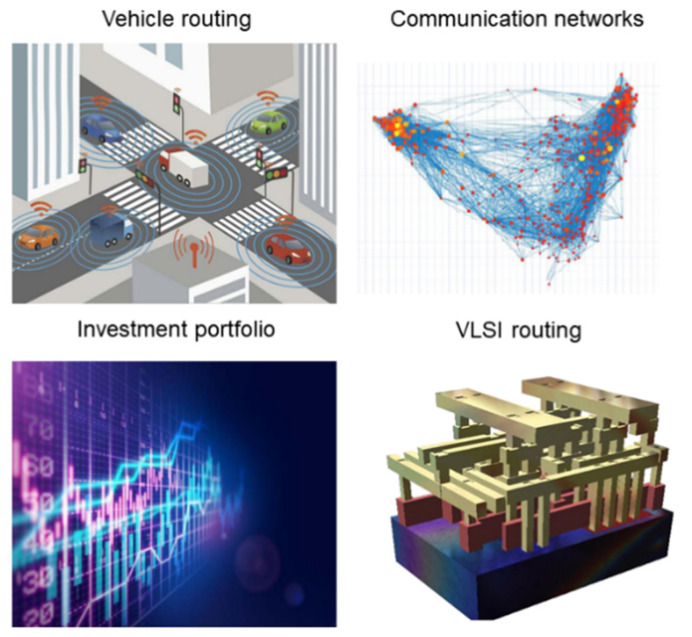
Applications of combinatorial optimization problems in real life [3].

Combinatorial optimization (CO) is an important branch of operations research and algorithm theory that has a wide spectrum of applications in the real world, including artificial intelligence (AI), circuit wiring, information network, route planning [1,2,3], etc. (Figure 1). Many of these CO problems belong to nondeterministic polynomial (NP)-completeness or NP-hard problems. In fact, these kinds of problems can be mapped to the problems of finding the ground state of the Ising Hamiltonian equation. However, it is generally intractable to solve such problems with traditional digital computers. Besides, with the increase of the problem’s dimensionality, the required computing power and time to obtain the global optimal solution will scale up exponentially [2]. Thus, researchers are looking for effective solutions to large-scale CO problems in the last decades.

**Figure 2:**
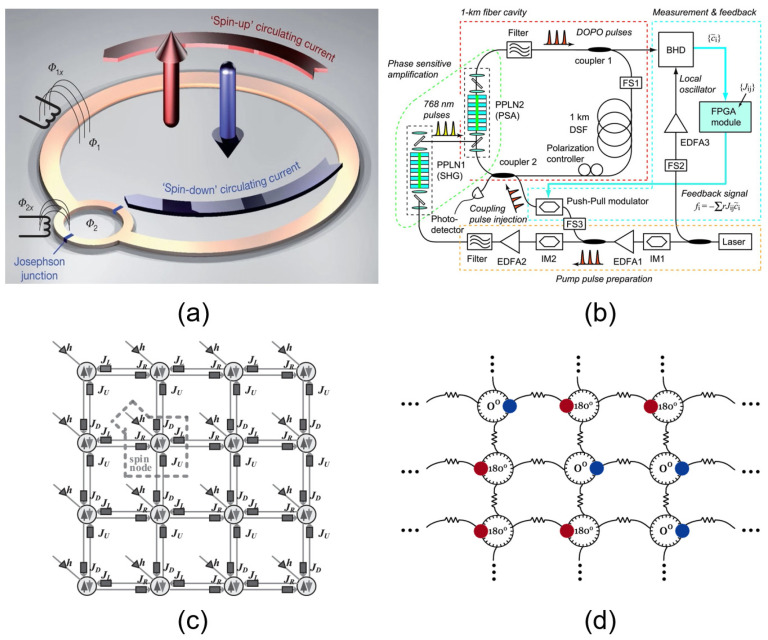
Schematic diagram of four different types of Ising machines: (**a**) quantum annealing Ising machine [5]; (**b**) optical coherent Ising machine [9]; (**c**) CMOS digital Ising machine [15]; (**d**) oscillator network Ising machine [18].

With the slowdown of Moore’s law, emerging hardware and computing paradigms beyond traditional transistor-based von-Neumann architecture are now topical. Different from the optimization algorithms tailored for digital logic circuits, Ising machines have the ability to solve the Ising Hamiltonian equation through their internal physical evolution process, which may yield an optimal solution at a high speed [4]. According to their physical mechanisms, existing Ising machines can be divided into four categories. The first one is the D-WAVE quantum annealing Ising machine [5,6,7,8] (Figure 2a), in which superconducting loops serve as spin nodes, and the nodes are coupled to each other through Josephson junctions. At the hardware level, the quantum annealing method has not demonstrated its advantages in real-world applications because this kind of machine can only work at ultra-low temperatures, which significantly increases the complexity and cost of the computing systems. The second type of Ising machine utilizes laser pulses as spins, and the coupling between spins is achieved through field-programmable gate arrays (FPGAs) [9,10,11,12] (Figure 2b). Although coherent optical Ising machines operate at room temperature, they also have a critical limitation that several kilometers of optical fiber ring cavity are required for Ising spins. This limits its application scenarios, and efforts are now being made toward system miniaturization and large-scale integration. Another promising approach is to implement Ising machines using the complementary metal oxide semiconductor (CMOS) digital circuits [13,14,15,16] (Figure 2c). Hardware accelerators using SRAM and FPGAs have also been widely studied to solve the Ising model. For example, a 20k-spin prototype Ising chip was reported by Yamaoka et al. The last is a coupled oscillator network–based Ising machine, which this paper will focus on. This type of Ising machine was first proposed by Wang et al. from UC Berkeley in 2017 [17,18], in which each oscillator acts as a spin node, and the spin state can be represented by the phase of the oscillator (Figure 2d). The nodes can be coupled through electrical, magnetic, or other effects. Compared with the first three kinds of Ising machines, Ising machines based on oscillator networks simultaneously hold the advantages of room-temperature operation, small size, low power consumption, and fast solution speed, which endows it with great potential in practical applications.

Thanks to these advantages of oscillator-network-based Ising machines, there is a recent surge of interest in this field. This paper has reviewed the development of this field for the first time, including the merits of different kinds of oscillators and the theoretical proof on why oscillator networks are equivalent to the corresponding Ising model and their performances. Finally, methods to improve the performance of this kind of solver are also discussed.

## 2. Theoretical Background

### 2.1. Types of Oscillators

In general, a physical system that produces periodic outputs can be termed an oscillator. In electronics, oscillators are usually regarded as a circuit component that outputs a periodic analog electrical voltage or current signal under direct excitation, which has a wide spectrum of applications such as clocking. According to the circuits and device physics, they can be divided into resistor–capacitor (RC) oscillators, inductor–capacitor (LC) oscillators, ring oscillators, phase-transition oscillators, spin torque oscillators, spin Hall oscillators, etc. In addition, according to the output waveforms, they can be categorized into three types: sine wave, square wave, or triangle wave oscillators. The performance of these Ising machines is highly relevant to the type of oscillators, which largely determines the physical footprint, time to solution, energy consumption, accuracy, and other important attributes of the solvers.

**Figure 3:**
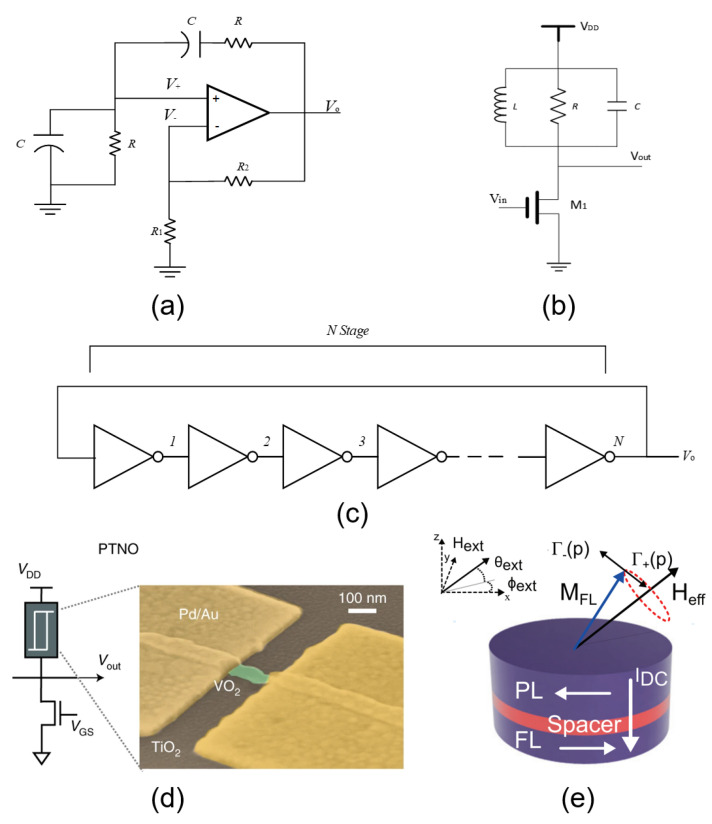
Schematic diagram of (**a**) RC oscillator; (**b**) LC oscillator; (**c**) ring oscillator; (**d**) phase-transition oscillator [22]; (**e**) spintronic oscillator [24].

A RC oscillator, that is, an oscillator composed of an RC frequency selection network (Figure 3a), is generally suitable for generating low-frequency oscillation signals below 1 MHz (oscillation frequency $f_{0}=\frac{1}{2\pi RC}$). Its key merit is that the circuit is simple and also easy to fabricate. However, in terms of the performance, its frequency selection capability is relatively limited. In addition, the output signal amplitude is not stable enough. Therefore, it is typically suitable for applications in which frequency stability is not required.

Like the RC oscillator, the LC oscillator is also very common, which employs a frequency selection network composed of LCs (Figure 3b) and leverages the energy storage characteristics of capacitors and inductors, alternating between these two types of electromagnetic energy. Compared with an RC oscillator, an LC oscillator is generally suitable for higher-frequency scenarios (oscillation frequency $f_{0}=\frac{1}{2\pi \sqrt{LC}}$). On one hand, increasing the oscillation frequency increases the electromagnetic power radiated by the LC circuit. On the other hand, because the frequency of the LC oscillator is negatively correlated with the size of the inductor, it needs a large footprint to meet the oscillation conditions at low frequency; as a result, a larger coil must be used. The high parasitic resistance of such a coil will lead to an increase in power consumption. In addition, the large-size inductor is also not favorable for miniaturization and integration.

A ring oscillator is a closed-loop oscillation circuit composed of an odd number of inverters (Figure 3c). Its oscillation frequency is determined by the inherent delay time of the gate circuit and the number of inverters (oscillation frequency $f_{0}=\frac{1}{2nt}$, where *t* is the delay time of a single inverter, *n* is the number of inverters). This kind of oscillators hold the advantages of simple circuitry and robust oscillation. The ring oscillator is one of the most compact transistor-based oscillators, which is highly conducive to large-scale integration. In addition, it also has decent energy consumption, which is comparable to that of nanoscale oscillators [19]. However, its disadvantage is that the oscillation frequency is not easy to modulate. In the absence of an external delay network, the oscillation frequency can only be tuned by changing the number of inverters.

Phase-transition oscillators, as the name suggests, are oscillators made of phase-transition materials (Figure 3d), which are a type of memristor oscillators [20,21]. The working principle is that there is a switch-like behavior in these phase-transition materials. When a current greater than the threshold passes through the phase-transition materials, it will cause a large and abrupt change in the conductivity. That is, under an applied electric field, the material undergoes a phase transition from an insulator to a conductor. Conversely, when the current is less than the threshold, a phase transition from conductor to insulator takes place. In addition, the phase-transition device can be combined with capacitors or resistors to implement relaxation oscillators [20,22]. Moreover, there is hysteresis in the phase-transition process due to the presence of the intrinsic electronic switching time, which has a significant influence on the oscillation frequency. This phase-transition material–based oscillator features a simple structure, a compact footprint, and low power consumption.

Spin torque and spin Hall oscillators (Figure 3e) are a new class of nanospintronic devices with promising applications in information storage, processing, and communication [23,24]. Its working principle mainly relies on the oscillating magnetic moment of the ferromagnetic material. Due to the tunnel magnetoresistance effect, the oscillation of the magnetic moment modulates the resistance of the magnetic tunneling layer, resulting in periodic oscillations of the output electrical signal. The energy required for these oscillations is provided by spin-polarized currents flowing through the magnetic layers, typically in the sub-mA level. Its oscillation frequency can be easily scaled up to the gigahertz range. Compared with semiconductor-based oscillators, it provides greater tunability, smaller size, lower power consumption, and higher integration density, showing great potential in high-speed and high-density computing. It is also one of the most popular physical implementations of neuromorphic circuits [25].

### 2.2. Ising Model and Max-Cut Problem

**Figure 4:**
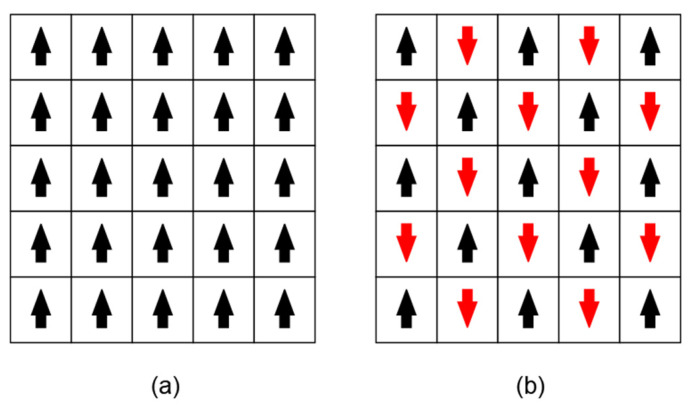
Schematic illustration of spin configuration in a (**a**) ferromagnetic system; (**b**) antiferromagnetic system.

The Ising model is a mathematical model named after the German physicist Ernst Ising. As early as the 1920s, the Ising model was proposed to describe the formation of magnetic domains in ferromagnets [26]. It contains a set of discrete variables, $s_{i}$, that describe the magnetic moment, also known as spin whose value is −1 or +1 to represent spin down or up, respectively. These magnetic moments are usually arranged by accounting for the interaction between adjacent spins. The “energy function” (Ising Hamiltonian equation) of the entire system can be written as follows:

(1) $$H=-\sum_{1\leq i\leq j\leq n}J_{ij}s_{i}s_{j}-\sum_{i=1}^{n}h_{i}s_{i}$$

where *n* represents the number of spins, $J_{ij}$ is the coupling coefficient between adjacent spins, which describes the polarity and magnitude of the interaction. For example, for each pair of spins *i* and *j*, if $J_{ij}>0$ or a ferromagnetic system, the energy of adjacent spins in the same state is lower, so the spins tend to be aligned in the same direction (Figure 4a). If $J_{ij}<0$, the system is antiferromagnetic; the energy of the adjacent spins in the opposite direction is lower, so the spins tend to be aligned in the opposite directions (Figure 4b). $J_{ij}=0$ indicates that there is no interaction between spins. $h_{i}$ represents the strength of the external magnetic field applied to each spin. For some specific problems (e.g., max-cut problem), a common simplification is to assume that there is no applied magnetic field, that is, $h_{i}=0$. Using this simplification, the Ising Hamiltonian equation can be written as

(2) $$H=-\sum_{i,j,i<j}J_{ij}s_{i}s_{j}$$

In fact, many CO problems have been proven to root on this equation. With the appropriate coupling coefficients $J_{ij}$, all of Karp’s 21 NP-complete problems can be translated to the equivalent Ising Hamiltonian equations [27,28]. The optimal solution is represented by the spin configuration with the lowest energy *H*_min_.

**Figure 5:**
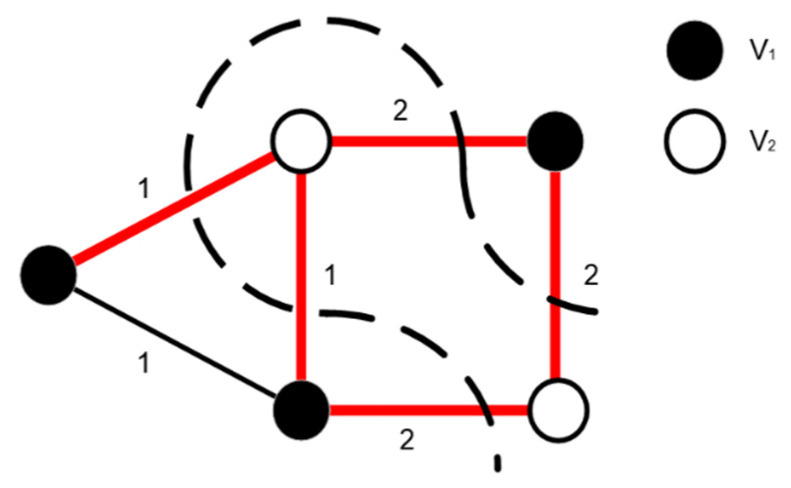
Schematic diagram of the max-cut problem.

The max-cut problem, for example (Figure 5), refers to finding a way to partition a given weighted graph into two sub-graphs to maximize the sum of the weights of all edges across two vertex sets (*V*_1_, *V*_2_) [29]. This is because all edges can be divided into three categories, namely the group connecting vertices in set *V*_1_, the group connecting vertices in set *V*_2_, and the group linking vertices in *V*_1_ and those in *V*_2_. The sum of the weights in these three sets are represented by *S*_1_, *S*_2_, and *S*_cut_, respectively. By setting $J_{ij}$ as the negative value of the edge weight between vertices *i* and *j*, we can get

(3) $$S_{1}+S_{2}+S_{cut}=\sum_{i,j,i<j}w_{ij}=-\sum_{i,j,i<j}J_{ij}$$

The vertices in two sets can be mapped to different spin values. For example, if *i*∈*V*_1_, then $s_{i}=+1$, if *i*∈*V*_2_, then $s_{i}=-1$. Then, the above Ising Hamiltonian equation can be rewritten as [30] follows:

(4) $$\begin{array}{l} H=-\sum_{i,j,i<j}J_{ij}s_{i}s_{j}\\ =-\sum_{i<j,ij\in V_{1}}J_{ij}\left(+1\right)\left(+1\right)-\sum_{i<j,ij\in V_{2}}J_{ij}\left(-1\right)\left(-1\right)-\sum_{i<j,i\in V_{1},j\in V_{2}}J_{ij}\left(+1\right)\left(-1\right)\\ =-\sum_{i<j,ij\in V_{1}}J_{ij}-\sum_{i<j,ij\in V_{2}}J_{ij}+\sum_{i<j,i\in V_{1},j\in V_{2}}J_{ij}\\ =S_{1}+S_{2}-S_{cut}\\ =\sum_{i,j,i<j}w_{ij}-2S_{cut} \end{array}$$

Therefore, when *H* is minimized, the sum of the cutting weights will be maximized. It should be noted that, for the max-cut problem, if the weight of the edge is positive, the mapped Ising model should be an antiferromagnetic system, that is $J_{ij}<0$. If the edge weight is negative, the mapped Ising model is ferromagnetic, i.e., all spins tend to be in the same state and the number of edges that have been cut remains 0.

### 2.3. The Formulation

**Figure 6:**
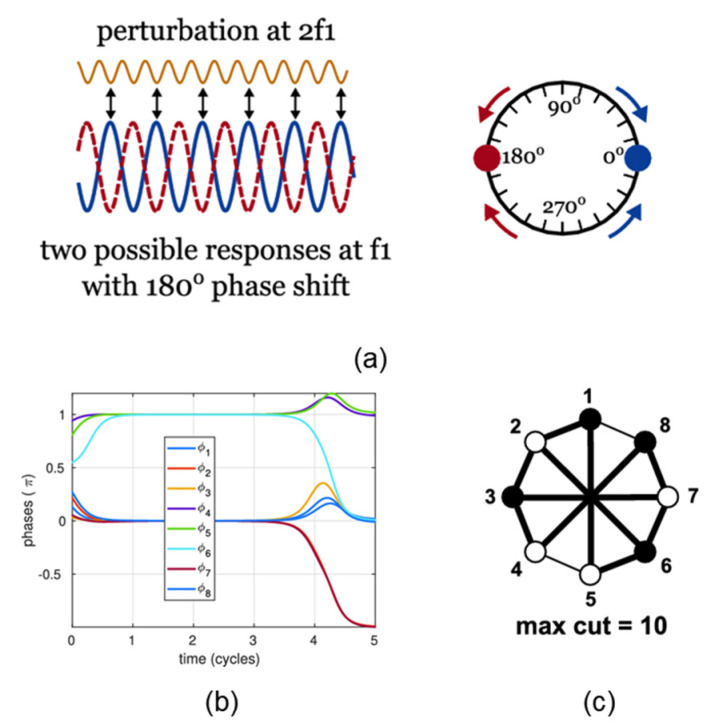
(**a**) Schematic diagram of second-harmonic injection locking (SHIL); (**b**) binarization of phases; (**c**) one of the optimal solutions to the 8-vertex max-cut problem [18].

This section introduces the theoretical formulation of a coupled oscillator network–based Ising machine and shows how the coupled oscillator networks automatically minimizes the Ising energy to yield the optimal solution. The implementation is mainly based on the fundamental injection locking (IL) and its variant second-harmonic injection locking (SHIL) [31,32,33,34] (these two phenomena will be discussed in detail subsequently). When an oscillator is perturbed by an external periodic signal whose frequency is almost twice the base oscillation frequency, then the oscillator’s phase will be in one of the two steady states, and the phase difference between the two states is 180° [34]. The spin state of each node in the Ising model can be encoded into the oscillator’s phase, using the two steady states that represent spin down or up, respectively. When these oscillators are coupled together in a certain manner, they will affect each other. Since the coupled oscillator network will automatically lower its total energy, the inherent physical evolution of the system will automatically solve the Ising Hamiltonian equation. The final steady-state phases of these oscillators represent the optimal solution (Figure 6).

We will first mathematically derive the dynamics of a single oscillator. IL is a nonlinear phenomenon that exists in oscillator systems. The term stands for the fact that the phase of the oscillator will be pulled or locked by an external periodic perturbation signal. When the frequency of the external signal is close to the oscillation frequency, this phenomenon is called IL. If the frequency of the external signal is nearly twice the oscillation frequency, then it is called SHIL.

A nonlinear oscillator is an autonomous dynamic system; when the oscillator is influenced by an external perturbation signal $\vec{b}\left(t\right)$, it can be described by the following differential algebraic equation (DAE) [35]:

(5) $$\frac{d}{dt}\vec{q}\left(\vec{x}\left(t\right)\right)+\vec{f}\left(\vec{x}\left(t\right)\right)=\vec{b}\left(t\right)$$

where $\vec{q}\left(\cdot \right)$ and $\vec{f}\left(\cdot \right)$ represent the nonlinear differential and algebraic parts, respectively. If the external perturbation is small, the solution, ${\vec{x}}_{p}\left(t\right)$, in Equation (5) can be simplified as [36]

(6) $${\vec{x}}_{p}\left(t\right)={\vec{x}}_{s}\left(t+\alpha \left(t\right)\right)$$

In which, ${\vec{x}}_{s}\left(t\right)$ with a period *T*_0_ is the steady-state solution without any perturbation, and $\alpha \left(t\right)$ represents the phase shift caused by the external signal and satisfies the following scalar equation:

(7) $$\frac{d}{dt}\alpha \left(t\right)={\vec{v}}_{1}^{T}\left(t+\alpha \left(t\right)\right)\cdot \vec{b}\left(t\right)$$

Here, ${\vec{v}}_{1}^{T}$ is the perturbation projection vector (PPV) of the oscillator. PPV has the same period as $\vec{x}\left(t\right)$ and is an inherent characteristic of the oscillator. It describes the sensitivity of the oscillator phase to a perturbation signal. The PPV of different types of oscillators has been discussed in the literature [33,37].

Assuming that the oscillation frequency is $f_{0}$, we define a PPV with 1-period

(8) $${\vec{v}}_{1}^{T}\left(t\right)=\vec{\chi }\left(f_{0}t\right)$$

When injecting an external periodic signal with a frequency $f_{1}$ ($f_{1}\approx f_{0}$), Equation (7) can be rewritten as

(9) $$\frac{d}{dt}\alpha \left(t\right)=\vec{\chi }\left(f_{0}\left(t+\alpha \left(t\right)\right)\right)\cdot \vec{b}\left(f_{1}t\right)$$

The phase difference between the oscillator and the external signal is defined as

(10) $$\Delta \phi \left(t\right)=\phi_{0}\left(t\right)-\phi_{1}\left(t\right)=f_{0}\left(t+\alpha \left(t\right)\right)-f_{1}t$$

Then, we have

(11) $$\alpha \left(t\right)=\frac{\Delta \phi \left(t\right)}{f_{0}}+\frac{f_{1}-f_{0}}{f_{0}}t$$

Combining Equations (9) and (11), we obtain

(12) $$\frac{d}{dt}\alpha \left(t\right)=\vec{\chi }\left(f_{0}\left(t+\alpha \left(t\right)\right)\right)\cdot \vec{b}\left(f_{1}t\right)=\frac{1}{f_{0}}\frac{d}{dt}\Delta \phi \left(t\right)+\frac{f_{1}-f_{0}}{f_{0}}$$

Combining Equation (10), we derive

(13) $$\frac{d}{dt}\Delta \phi \left(t\right)=-\left(f_{1}-f_{0}\right)+f_{0}\vec{\chi }\left(\Delta \phi \left(t\right)+\phi_{1}\left(t\right)\right)\cdot \vec{b}\left(\phi_{1}\left(t\right)\right)$$

In Equation (13), we assume that $\phi_{1}\left(t\right)$ is a “fast” varying variable and $\Delta \phi \left(t\right)$ is a “slowly” varying variable. Averaging the “fast” varying $\phi_{1}\left(t\right)$ and retaining the “slow” varying $\Delta \phi \left(t\right)$, we define:

(14) $$g\left(\Delta \phi \left(t\right)\right)=\frac{1}{T_{1}}\int_{0}^{T_{1}}\chi \left(\Delta \phi \left(t\right)+\phi_{1}\left(t\right)\right)\cdot b\left(\phi_{1}\left(t\right)\right)d\phi_{1}\left(t\right)$$

Then,

(15) $$\frac{d}{dt}\Delta \phi \left(t\right)=-\left(f_{1}-f_{0}\right)+f_{0}g\left(\Delta \phi \left(t\right)\right)$$

Equation (15) is the generalized Adler’s equation describing the IL phenomenon [33].

Taking the LC oscillator as an example, when an LC oscillator (PPV: $\vec{\chi }\left(t\right)=-\sqrt{\frac{L}{C}}\frac{1}{A}\sin\left(f_{0}t\right)$) is disturbed by a sinusoidal signal, Equation (15) is transformed to the Adler’s equation [38]:

(16) $$\frac{d}{dt}\Delta \phi \left(t\right)=-\left(f_{1}-f_{0}\right)-\frac{I_{1}}{I_{0}}\frac{f_{0}}{2Q}\sin\left(\Delta \phi \left(t\right)\right)$$

Here, $I_{1}$ represents the strength of the external signal, $I_{0}$ is the strength of the oscillator output current, and *Q* is the quality factor of the oscillator.

When several oscillators are coupled together, the oscillator of interest is subject to the injection of output signals of other oscillators according to Equation (16):

(17) $$\frac{d}{dt}\left(\phi_{i}\left(t\right)-\phi_{j}\left(t\right)\right)=\frac{d}{dt}\left(\phi_{i}\left(t\right)-f_{j}t\right)=-\left(f_{j}-f_{i}\right)-\frac{1}{N}\sum_{j=1}^{N}K_{ij}\sin\left(\phi_{i}\left(t\right)-\phi_{j}\left(t\right)\right)$$

Then,

(18) $$\frac{d}{dt}\phi_{i}\left(t\right)=f_{i}-\frac{1}{N}\sum_{j=1}^{N}K_{ij}\sin\left(\phi_{i}\left(t\right)-\phi_{j}\left(t\right)\right)$$

Equation (18) is known as the Kuramoto model [39], where *N* is the number of oscillators and $K_{ij}$ represents the coupling coefficient between the oscillators.

In addition, when the oscillator is locked by an external signal, $\Delta \phi \left(t\right)$ will become a constant, $\frac{d}{dt}\Delta \phi \left(t\right)=0$, according to Equation (15):

(19) $$\frac{f_{1}-f_{0}}{f_{0}}=g\left(\Delta \phi \left(t\right)\right)$$

From Equation (19), the range of IL and the phase difference between the oscillators can be easily determined.

The total energy is determined by the Lyapunov equation [40]:

(20) $$\frac{d\Delta \phi (t)}{dt}=-\frac{\partial E}{\partial \Delta \phi (t)}$$

The derivative of energy with respect to time is

(21) $$\frac{\partial E}{\partial t}=\frac{\partial E}{\partial \Delta \phi \left(t\right)}\frac{d\Delta \phi \left(t\right)}{dt}=-{\left(\frac{d\Delta \phi \left(t\right)}{dt}\right)}^{2}\leq 0$$

From Equation (21), it can be seen that this system has a tendency to reduce the energy over time automatically.

**Figure 7:**
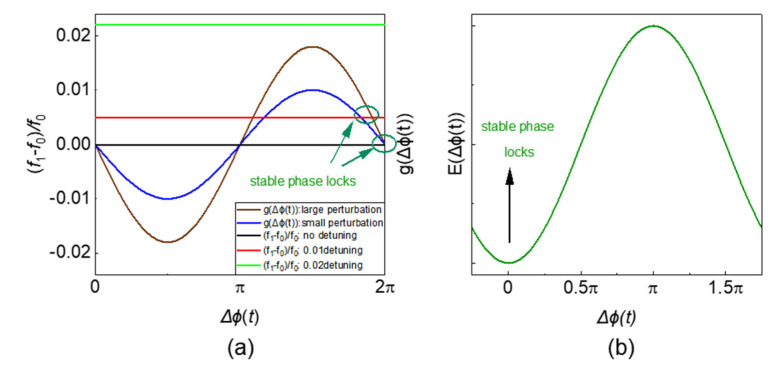
(**a**) Graphical solutions of first-order injection locking range and phase; (**b**) relationship between system’s energy and phase difference.

When $\frac{d\Delta \phi \left(t\right)}{dt}=0$, $\frac{\partial E}{\partial \Delta \phi (t)}=0$, the period of $g\left(\Delta \phi \left(t\right)\right)$ in Figure 7a is 2π. There are two intersection points representing a maximum value and a minimum value of the system energy in Figure 7b. However, which point represents the steady (or unsteady) state depends on the polarity of the injected signal (coupling coefficient between oscillators).

Assuming that there are two LC oscillators with the same oscillation frequency,

(22) $$E\left(\Delta \phi (t)\right)=-\frac{1}{2}K_{ij}\cos\left(\phi_{i}\left(t\right)-\phi_{j}\left(t\right)\right)$$

**Figure 8:**
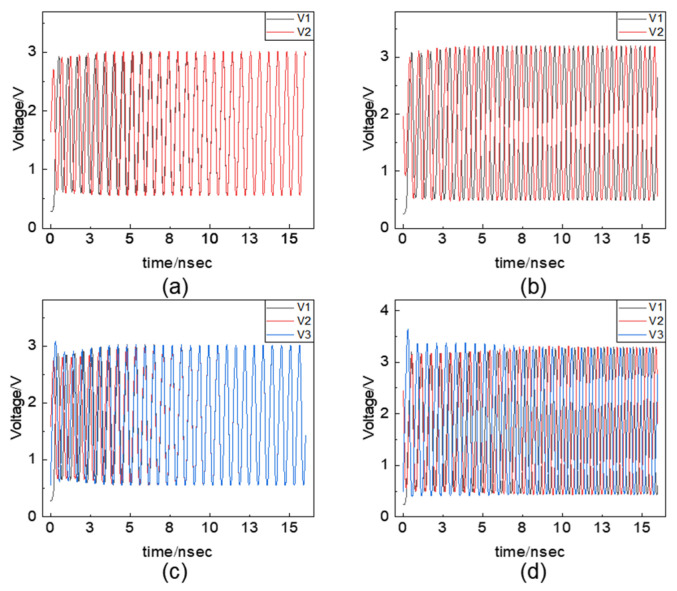
Phase diagram of (**a**) 2 positively coupled oscillators; (**b**) 2 negatively coupled oscillators; (**c**) 3 positively coupled oscillators; (**d**) 3 negatively coupled oscillators.

It can be seen from Equation (22) that, if $K_{ij}$ is positive, which means that there is a positive coupling between oscillators, the system’s energy reaches the minimum when $\phi_{i}\left(t\right)-\phi_{j}\left(t\right)=0$, and their phases will tend to be the same (Figure 8a). However, if $K_{ij}$ is negative, which means that there is a negative coupling between oscillators, the energy takes the minimum value when $\phi_{i}\left(t\right)-\phi_{j}\left(t\right)=\pi$, and their phases will tend to be opposite (Figure 8b). When multiple oscillators are interfaced to each other through negative coupling, the phase of each oscillator and its neighbors tend to be opposite, and their phases cannot stay binary (0/π) under mutual interaction (Figure 8d). In this case, SHIL is required.

When the oscillator is subjected to an external perturbation signal of about twice the base frequency ($f_{1}\approx 2f_{0}$), the generalized SHIL Adler’s equation is [34]

(23) $$\frac{d}{dt}\Delta \phi \left(t\right)=-\left(\frac{1}{2}f_{1}-f_{0}\right)+f_{0}g\left(\Delta \phi \left(t\right)\right)$$

In which,

(24) $$g\left(\Delta \phi \left(t\right)\right)=\frac{1}{2T_{1}}\int_{0}^{2T_{1}}\chi \left(\Delta \phi (t)+\frac{1}{2}\phi_{1}\left(t\right)\right)\cdot b\left(\phi_{1}\left(t\right)\right)d\phi_{1}\left(t\right),\Delta \phi \left(t\right)=\phi \left(t\right)-\frac{1}{2}\phi_{1}\left(t\right)$$

In order to study its periodicity, the PPV and injection signal were expanded using Fourier series:

(25) $$\chi \left(\Delta \phi \left(t\right)+\frac{1}{2}\phi_{1}\left(t\right)\right)=\sum_{k=-\infty }^{\infty }\chi_{k}e^{j2\pi k\left(\Delta \phi \left(t\right)+\frac{1}{2}\phi_{1}\left(t\right)\right)},\;b\left(\phi_{1}\left(t\right)\right)=\sum_{l=-\infty }^{\infty }b_{l}e^{j2\pi l\phi_{1}\left(t\right)}$$

Then,

(26) $$g\left(\Delta \phi \left(t\right)\right)=\sum_{l=-\infty }^{\infty }\chi_{-2l}b_{l}e^{-j2\pi 2l\Delta \phi \left(t\right)}$$

where χ and b are the Fourier coefficients. From Equation (26), we know that the period of $g\left(\Delta \phi \left(t\right)\right)$ is half of the oscillator’s period. When locked by the injection signal, $\frac{d}{dt}\Delta \phi \left(t\right)=0$, then, we have

(27) $$\frac{\frac{1}{2}f_{1}-f_{0}}{f_{0}}=g\left(\Delta \phi \left(t\right)\right)$$

Assuming that $f_{1}=2f_{0}$, according to the Lyapunov equation, the total energy of the system is

(28) $$E\left(\Delta \phi \left(t\right)\right)=f_{0}\sum_{l=-\infty }^{\infty }\frac{\chi_{-2l}b_{l}}{j2\pi 2l}e^{-j2\pi 2l\Delta \phi \left(t\right)}$$

**Figure 9:**
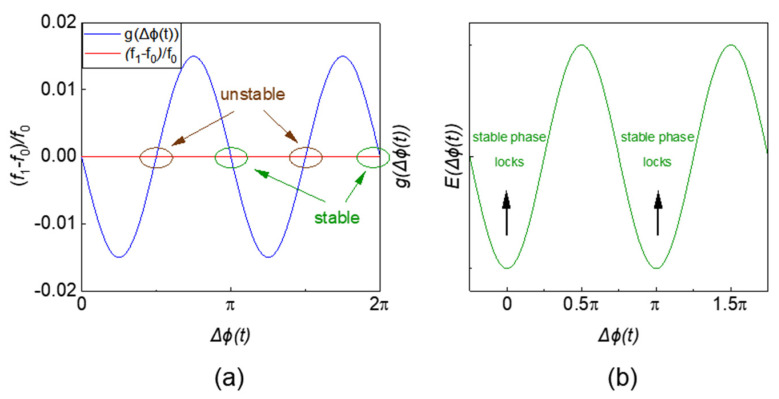
(**a**) Graphical solutions of the locking phase and range of SHIL; (**b**) the relationship between the system’s energy and phase difference.

It can be seen from Figure 9a that there are 4 intersection points, which represent the two maxima and two minima of the system energy in Figure 9b, respectively. The phase difference between the two minima is π.

When an oscillator network is perturbed by an injection signal with a frequency of $f_{1}$, where $f_{1}=2f_{0}$, each oscillator will be simultaneously affected by the external signal and the output signals of the other oscillators. Supposing that the oscillator network has a consistent frequency and that the LC oscillators are perturbed by a sinusoidal signal, then,

(29) $$\frac{d\phi_{i}\left(t\right)}{dt}=-\frac{K_{ij}}{N}\sum_{j=1}^{N}\sin\left(\phi_{i}\left(t\right)-\phi_{j}\left(t\right)\right)-K_{s}\sin\left(2\left(\phi_{i}\left(t\right)-\phi_{1}\left(t\right)\right)\right)$$

The system’s energy is

(30) $$E=-\frac{K_{ij}}{N}\sum_{j=1}^{N}\cos\left(\phi_{i}\left(t\right)-\phi_{j}\left(t\right)\right)-\frac{1}{2}K_{s}\cos\left(2\left(\phi_{i}\left(t\right)-\phi_{1}\left(t\right)\right)\right)$$

where $K_{s}$ represents the relative strength of the externally injected signal. From the above analysis, when the oscillator is locked by an external signal with double frequency, $\phi_{i}\left(t\right)-\phi_{1}\left(t\right)=0/\pi$, then $\phi_{i}\left(t\right)-\phi_{j}\left(t\right)=0/\pi$. In addition, let $K_{ij}=NJ_{ij}$, then,

(31) $$E=-\sum_{i,j,i\neq j}^{N}J_{ij}\cos\left(\phi_{i}\left(t\right)-\phi_{j}\left(t\right)\right)-\frac{1}{2}NK_{s}$$

**Figure 10:**
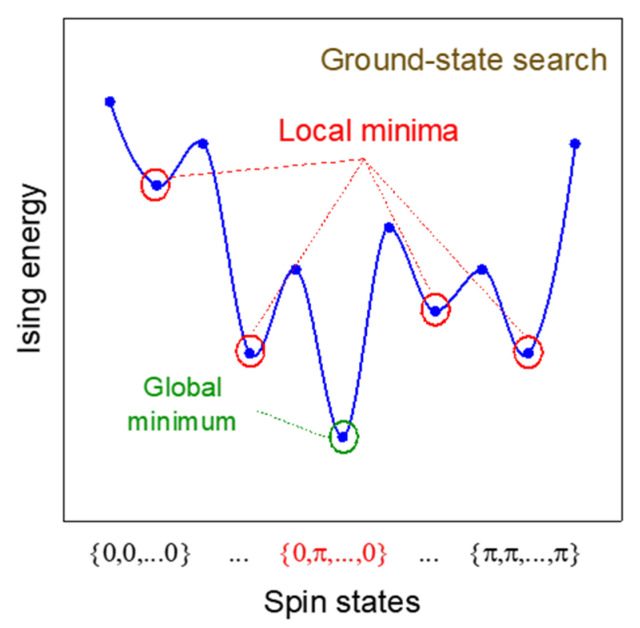
Schematic diagram of oscillator system energy.

Equation (31) is very similar to the original Ising Hamiltonian equation. When $\phi_{i}\left(t\right)-\phi_{j}\left(t\right)=0$, $s_{i}s_{j}=-1$, while $\phi_{i}\left(t\right)-\phi_{j}\left(t\right)=\pi$, $s_{i}s_{j}=-1$. What should be emphasized is that even under the influence of the injection signal with twice the frequency, when $K_{ij}$ ($J_{ij}$) is positive or the coupling between oscillators is positive, the system’s energy reaches the minimum when $\phi_{i}\left(t\right)-\phi_{j}\left(t\right)=0$. Therefore, the phase of the coupled oscillators will still tend to be consistent, which is equivalent to a ferromagnetic system. If $K_{ij}$ is negative, there is a negative coupling between oscillators, the system is equivalent to an antiferromagnetic system and the phases of the connected oscillators will tend to be opposite. In addition, the system’s energy will automatically decrease with time, which proves that this system can be used as an Ising machine to automatically solve the Hamiltonian equation using its inherent physical evolution. In general, for a complex system, there are many local minima in this equation as shown in Figure 10. The system may fall into one of the local optimal solutions during the state evolution. Simply relying on this dynamic process cannot guarantee that the global optimal solution can be acquired. The system also needs some sort of help (for example, tunable noise) to escape from the local optimum. The annealing process, featuring a gradually decaying noise, will increase the probability of reaching the global optimum [18,22]. When design such Ising machines, in addition to the properties of the oscillators, we should also consider the coupling strength between oscillators as well as the strength of external injection signals and noise, which are the three main parameters that will affect the performance of this kind of Ising machine.

## 3. Experimental Demonstrations

**Figure 11:**
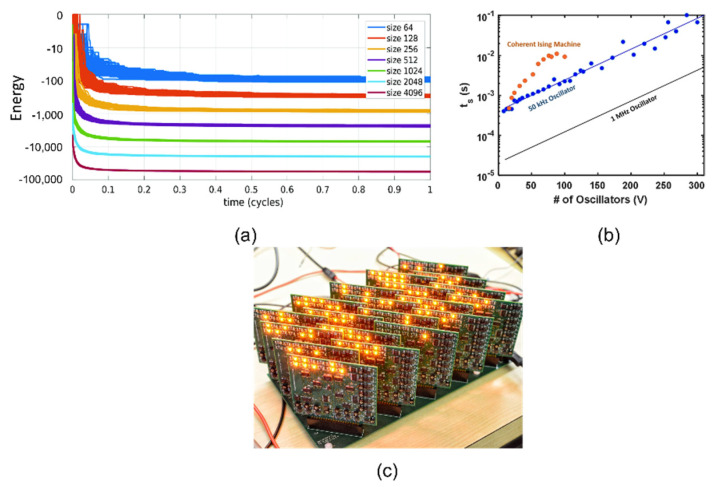
(**a**) Relationship between the convergence time of the Ising function and the size of the oscillator network [18]; (**b**) relationship between the solution time of the oscillator network and the size and oscillation frequency [41]; (**c**) 240 LC oscillator network [42].

In 2017, Wang et al. demonstrated for the first time that a self-sustaining LC oscillator network can be used as an Ising machine. They also showed how the global Lyapunov function of the oscillator phase macromodel can be mapped to the corresponding Ising equation [17,18]. Moreover, they also used this type of Ising machine to solve large-scale max-cut problems. Among the 54 G-set problems, 38 secured optimal solutions, illustrating its promising performance. It was also found that using different types of oscillators or injection signals with different waveforms may increase the probability of successfully obtaining the global optimal solution. However, increasing the frequency deviation between oscillators will degrade the performance. Fortunately, if such deviation is within a certain range, this shortcoming could be compensated for by using tunable oscillators or by increasing the coupling strength between oscillators. In addition, it is also very important to use an optimized noise annealing process. Compared with other types of Ising machines, this oscillatory Ising machine had a better solution quality and obtained more cutting weight in dealing with many max-cut problems. In addition, through theoretical analysis, it was also shown that the convergence rate of the system’s energy function remained unchanged upon increment of the problem size [18] (Figure 11a). In addition, the solving speed was faster than that of other solvers, which makes it advantageous for solving large-scale CO problems. However, it should be noted that, in practice, the total computation time is also related to the annealing process. Since increasing the problem size will increase the number of local minima, the solution time may also increase [22,41] (Figure 11b). The computation time is mainly determined by the oscillation frequency; therefore, increasing the oscillation frequency is beneficial for improving the solving speed [17,18,41]. In terms of hardware implementation, Wang et al. demonstrated an inverter cross-coupled LC oscillator network, physically realizing a prototype Ising machine with up to 240 spin nodes and programmability [42] (Figure 11c). As for this machine, the oscillation frequency was about 1 MHz, and the power consumption of the whole device was about 5 W. The programmability was realized by an adjustable digital potentiometer, and male and female pin connectors were used to control the coupling polarity. This physical Ising machine prototype successfully solved several randomly generated Ising problems, and the measured performance was better than that of the best algorithm solver SDP. In a similar work, a network consisting of four fully coupled LC oscillators was also demonstrated [41]. However, the low oscillation frequency and large physical dimension of LC oscillators are not conducive to system integration.

**Figure 12:**
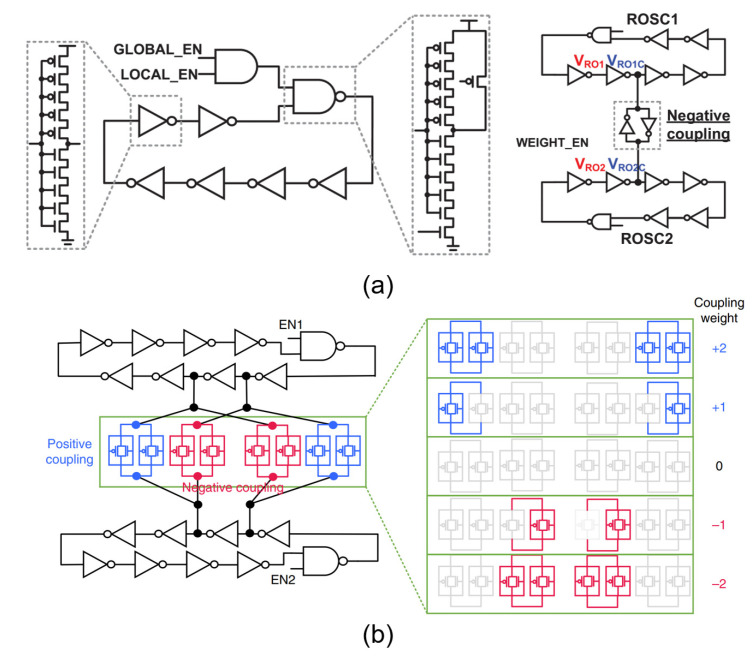
(**a**) Ring oscillator network coupled through inverters [3]; (**b**) same-node and cross-node coupling configuration for positive and negative coupling, transmission gates controlled five coupling strengths [44].

Compared with LC oscillators, ring oscillators can easily achieve higher working frequencies and can be easily integrated. Ahmed et al. fabricated a programmable network consisting of 560 coupled ring oscillators and successfully realized a fully integrated chip-scale Ising machine [3,43]. Each oscillator and its adjacent oscillators were negatively coupled using a set of anti-parallel connected inverters (Figure 12a), and the coupling coefficient could be easily adjusted by the control signal for real-time programming, which enables it to solve a variety of max-cut problems with different sizes. Experiments showed that the success rate of this Ising machine in solving max-cut problems was as high as 82–100%. Compared with commercial software, the solving speed was 10^4^–10^6^ times faster, while the power consumption of the entire Ising machine was only 23 mW, corroborating its great potential for practical applications.

Recently, Moy et al. reported a 1968-node coupled King’s graph ring oscillator–based Ising machine with five-level coupling strengths [44]. Here, the programmability was implemented by the transmission gates. Each gate could be separately turned on/off to enable the five coupling states. In addition to using inverters, the negative coupling weight could also be realized by the cross-coupling configuration because of the 180° phase difference between the adjacent nodes of the ring oscillators (Figure 12b). This Ising chip has excellent performance as it features a computation accuracy over 95% for randomly generated CO problems with an average power of 42 mW and an overall computation time for the global optimal solution of only 50 ns. 

**Figure 13:**
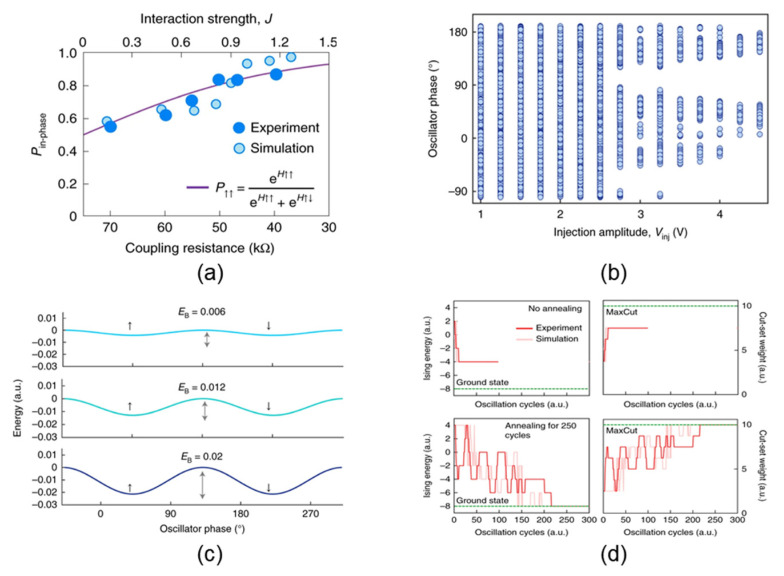
(**a**) Relationship between the synchronization probability and coupling strength; (**b**) relationship between the phase value and injection locking strength; (**c**) relationship between energy barrier height and injection locking strength; (**d**) comparison of the Ising machine’s performance with and without annealing processes [22].

Dutta et al. developed a phase-transition nano-oscillator network using VO_2_ memristors and investigated the impact of the coupling coefficients, the power of injection signals, and the annealing noise on solver’s performance [22,45,46]. It was found that enhancing the coupling strength between these oscillators would increase the energy exchange efficiency, thereby increasing the success probability of synchronization (Figure 13a). To ensure that the oscillator network has a binarized phase, the intensity of the injected disturbance signal should be greater than a certain threshold, below which a stable solution cannot exist (Figure 13b). This is due to the fact that the phases of the oscillators will fluctuate randomly under the influence of noise, so the strength of the injected signal will affect the energy barrier between the energy minima at the same time. When there is a weak injection perturbation, they can easily jump out from the minimum, and the phases cannot be locked to the lowest energy state under the influence of noise. When the intensity of the injection signal increases, the height of the energy barrier between the minima also increases (Figure 13c); this will weaken the influence of noise and keep the states more stable. However, on the other hand, the intensity should not be too large. This is because when the energy barrier is too large, the state or phases may be trapped in a local minimum and will not have enough energy to escape, resulting in a “freeze-out effect”, which is not favorable for the system to reach the ground state [46]. In addition, the authors also employed the annealing process to effectively improve the success probability (Figure 13d). With a progressively increasing intensity of the perturbation signal and a decreasing noise, the probability of the system reaching the ground state will greatly increase [22]. Therefore, this work suggests that the system design should take the coupling strength between oscillators and the power of the injected signal and noise into consideration.

**Figure 14:**
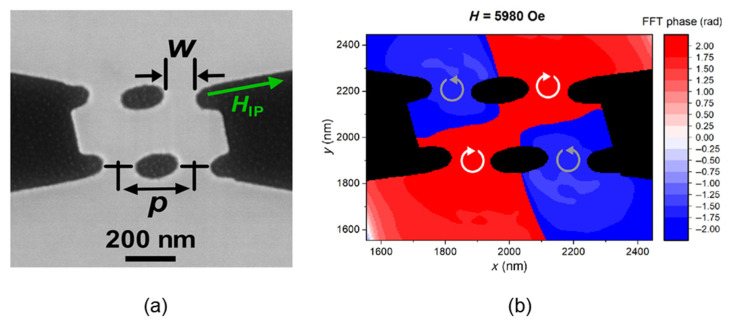
(**a**) SEM image of the 2 × 2 spin Hall nano-oscillator array; (**b**) phase image of the 2 × 2 spin Hall nano-oscillator array [52].

In terms of novel nanospintronic oscillators, McGoldrick et al. theoretically analyzed the performance of the Ising machine based on the electrically coupled spin Hall nano-oscillators and established a theoretical model to describe the process of IL phenomenon based on spin Hall oscillators [47]. They also pointed out that its computation speed could be reduced to the nanosecond scale thanks to the increase of the oscillator frequency. In addition to using electrical coupling, nanospintronic oscillators can also couple through the interaction of spin waves or magnetic dipoles [48,49,50,51]. Houshang et al. reported a 2 × 2 spin Hall nano-oscillator array (Figure 14) coupled through magnetic dipoles, which successfully solved several max-cut problems [52]. Direct coupling can simplify the network structure to a large extent, and it is also beneficial for system footprint reduction. However, due to the attenuation of spin waves during propagation and the limited propagation distance, an oscillator can only couple with the adjacent oscillators, and the coupling strength between them is not easy to modulate [48]. Thus far, it is still difficult to achieve global coupling through direct coupling, and the number of oscillators is also limited.

Table 1 compares and summarizes the performance of different types of oscillator-network-based Ising machines. Overall, it can be seen that the solving speed of this kind of Ising machines is relatively fast, and the time to produce optimal solution can be scaled down to nanoseconds with an overall power consumption of a few milliwatts. In addition, programmable coupling weights make oscillator-network-based Ising machines capable of solving different CO problems. However, in terms of hardware implementation, the number of oscillators is still relatively small. 

## 4. Conclusions

Thanks to the unique advantages of these oscillator-network-based Ising machines in solving intractable CO problems, this research area is receiving increasing attention. In this paper, the research progress in this field was surveyed for the first time. Firstly, the pros and cons of different types of oscillators were discussed. Secondly, the theoretical formulation of the Ising model was derived, and the mapping between the coupled oscillator networks under SHIL and the Ising machine for solving max-cut problem was discussed. Last, the implementation and performance of the existing oscillator-network-based Ising machines were summarized. 

Compared with other types of Ising solvers, oscillator-network-based Ising machines have shown their advantages in fast solving speed and low power consumption. Although many breakthroughs have been made in this direction, the oscillator-network-based Ising machines are still relatively primitive compared to well-developed digital systems in terms of performance, hardware cost, and complexity. Performance-wise, there is large room for improvement. For example, the dynamic properties of oscillators have a profound influence on the performance of the Ising machine. Thus, there is a constant pursuit for faster, more compact, and less noisy oscillators. Hardware cost-wise, CMOS-based oscillators are relatively expensive in terms of fabrication. A possible solution is to employ emerging memristive nanoscale oscillators. However, such oscillators may suffer from intrinsic stochasticity, making it difficult to keep the oscillating frequency of memristive nanoscale oscillators in the network precisely the same using conventional fabrication techniques. Such frequency deviation may significantly impact the system performance by degrading the success rate of hitting the global optimum. Hardware complexity-wise, the current coupling weights are either not reconfigurable (e.g., capacitors) or bulky (e.g., external potentiometer). Implementing programmable weights using nonvolatile memristors is a promising solution to reduce the physical system footprint and idle power, while adapting to different tasks. Furthermore, small-scale prototypical hardware demonstrations based on different types of oscillators have proved this concept, while large-scale implementations are still rare and mostly simulation based.

As CO problems are quickly growing in the era of Big Data and Internet of Things (IoT), novel hardware Ising solvers are of great interest to both academia and industry. In the near future, coupled oscillator network–based Ising machines with the advantages of high integration density, low power consumption, and fast solving speed are likely to have a wide spectrum of applications in mobile edge devices and data centers, which are expected to have a transformative impact on the computing technology.

## Figures and Tables

**Table 1:** Performance of different types of oscillator-network-based Ising machines.

Type of Oscillator	Oscillation Frequency	Number of Nodes	Coupling Method	Coupling Weight	Solution Time	Power Consumption	Success Probability	Reference
LC	1 MHz	240	R	Programmable	1 ms	5 W	/	[42]
LC	50 kHz	4	R	Programmable	100 μs	/	98%	[41]
Ring	118 MHz	560	Inverter	Programmable	200 ns	23 mW	82–100%	[3]
Ring	1 GHz	1968	Transmission gates	Programmable	50 ns	42 mW	89–100%	[44]
Schmitt-trigger	45 kHz	30	C	Programmable	/	1.76 mW	72%	[53]
VO_2_ phase-transition	500 MHz	8	C	No	30 μs	2.56 mW	96%	[22]
Spin Hall *	/	100	C	No	6.8 μs	11.5 mW	/	[47]
Spin Hall	7.8 GHz	4	Magnetic dipole	Programmable	/	144 mW	/	[52]

* Simulation result.
